# GATA2 Mediates Thyrotropin-Releasing Hormone-Induced Transcriptional Activation of the Thyrotropin β Gene

**DOI:** 10.1371/journal.pone.0018667

**Published:** 2011-04-14

**Authors:** Kenji Ohba, Shigekazu Sasaki, Akio Matsushita, Hiroyuki Iwaki, Hideyuki Matsunaga, Shingo Suzuki, Keiko Ishizuka, Hiroko Misawa, Yutaka Oki, Hirotoshi Nakamura

**Affiliations:** Second Division, Department of Internal Medicine, Hamamatsu University School of Medicine, Hamamatsu, Shizuoka, Japan; New Mexico State University, United States of America

## Abstract

Thyrotropin-releasing hormone (TRH) activates not only the secretion of thyrotropin (TSH) but also the transcription of TSHβ and α-glycoprotein (αGSU) subunit genes. TSHβ expression is maintained by two transcription factors, Pit1 and GATA2, and is negatively regulated by thyroid hormone (T3). Our prior studies suggest that the main activator of the TSHβ gene is GATA2, not Pit1 or unliganded T3 receptor (TR). In previous studies on the mechanism of TRH-induced activation of the TSHβ gene, the involvements of Pit1 and TR have been investigated, but the role of GATA2 has not been clarified. Using kidney-derived CV1 cells and pituitary-derived GH3 and TαT1 cells, we demonstrate here that TRH signaling enhances GATA2-dependent activation of the TSHβ promoter and that TRH-induced activity is abolished by amino acid substitution in the GATA2-Zn finger domain or mutation of GATA-responsive element in the TSHβ gene. In CV1 cells transfected with TRH receptor expression plasmid, GATA2-dependent transactivation of αGSU and endothelin-1 promoters was enhanced by TRH. In the gel shift assay, TRH signal potentiated the DNA-binding capacity of GATA2. While inhibition by T3 is dominant over TRH-induced activation, unliganded TR or the putative negative T3-responsive element are not required for TRH-induced stimulation. Studies using GH3 cells showed that TRH-induced activity of the TSHβ promoter depends on protein kinase C but not the mitogen-activated protein kinase, suggesting that the signaling pathway is different from that in the prolactin gene. These results indicate that GATA2 is the principal mediator of the TRH signaling pathway in TSHβ expression.

## Introduction

The hypothalamus–pituitary–thyroid (H-P-T) axis is the central mechanism for thyroid hormone (T3) homeostasis [1], [2]. Thyrotropin (TSH) is a heterodimer of the α chain (α subunit of glycoprotein hormone, αGSU) and β chain (TSHβ). TSHβ is specific to TSH while αGSU is common to luteinizing hormone, follicle-stimulating hormone and chorionic gonadotropin [3]. Secretion of the TSH molecule from thyrotroph and the transcriptions of TSHβ and αGSU genes are stimulated by the thyrotropin-releasing hormone (TRH) generated in the hypothalamic paraventricular nucleus. In rat pituitary primary culture, for example, TRH treatment increases αGSU and TSHβ mRNA [4], [5]. Conversely, TSHβ expression is attenuated in mice homologous for a TRH-null allele after birth [6]. TRH receptor (TRH-R) is encoded by two genes and generates TRH-R1 and 2. In the pituitary, TRH-R1 but not TRH-R2 is expressed and mediates the TRH signal [7]. TRH-bound TRH-R1 (TRH/TRH-R1) induces protein kinase C (PKC)-, phosphophatidyl-inoshitol- and Ca^2+^-mediated pathways [8], [9]. PKC subsequently potentiates multiple transcription factors, including Jun and Fos, via the AP-1 site [10]; however, the mechanism that mediates TRH signaling for transcription of the TSHβ gene has been elusive.

A pituitary-specific transcription factor, Pit1, has been postulated to be a candidate mediator of TRH signaling in TSHβ gene regulation. This possibility is supported by the fact that Pit1 mutations cause compound pituitary hormone deficiency (CPHD) [11], in which expressions of the TSHβ gene as well as the prolactin (PRL) and growth hormone (GH) genes are decreased or abolished. Using reporter assays with somato-lactotroph-derived GH3 [12] cells and gel shift assays, Shupnik et al. [13], [14] reported two Pit1 binding sites, TSH-A (nt. −274/−258) and C (nt. −402/−384) while Steinfelder et al. [15], [16] demonstrated other putative Pit1 binding sites within nt. −128/+8 of the human TSHβ gene. Although Gordon et al. [17] confirmed that Pit1 recognizes the DNA sequences in the mouse TSHβ gene corresponding to those sites in the rat and human genomes using DNA foot printing, overexpression of Pit1 had a minimal effect on the activity of the TSHβ promoter in TtT97 TSHoma or thyrotroph-derived αTSH cells. Similar results were reported with kidney-derived 293 cells over-expressed with Pit1 [18]. Consistently, TRH treatment does not enhance transcriptional activity of the fusion protein Gal4-Pit1, where the Pit1-derived transactivation domain was fused with the Gal4-DNA binding domain [19]. Although Pit1 may be phosphorylated at serine (codon 115) and threonine (codon 220) by PKA [20] or by TRH signaling [15], [16], mutations of these amino acids have no effect on its transactivation function in the PRL promoter [19], [21].

T3 inhibits transcription of the TSHβ gene via thyroid hormone receptor (TR) [1], and TSHβ expression increases in patients with hypothyroidism. This raises the possibility that unliganded TR may function as a transcriptional activator of the TSHβ gene [22]–[24]. Based on this hypothesis, the putative negative T3 responsive element (nTRE) has been reported as the sequence required for activation by unliganded TR and inhibition by T3 [22], [25]. Because an AP-1-like sequence [26] overlaps with the reported nTRE, it was suggested that interaction of unliganded TR with Jun/Fos may play a role in the TRH signaling pathway [27]; however, the findings that TSHβ expression can be maintained in TR-null mice [24], [28] rejected the possibility that unliganded TR is an activator, indicating the presence of another transcriptional activator [29].

As a TSHβ-related transcription factor that exists in TtT97 TSHoma cells but not in GH3 cells, Gordon et al. [30] identified GATA2. In vivo analysis using transgenic mice revealed that co-expression of GATA2 with Pit1 directs the differentiation of thyrotroph in the pituitary [3]. We reported that the true transcriptional activator for the TSHβ gene is GATA2 but not Pit1 and that Pit1 protects GATA2 from inhibition by the suppressor region (SR) located in the 3′-flanking region of GATA-REs [31]. We reported that when TR is coexpressed with GATA2 and Pit1, T3-dependent inhibition is easily detected in kidney-derived CV1 cells [32], which have been used in the studies of transcriptional activation by T3/TR (positive regulation) [29], [33], [34]. Establishment of this experimental system suggests that thyrotroph-specific factors other than Pit1 and GATA2 may not be essential for T3-dependent negative regulation of the TSHβ gene [29]. Indeed, repression by T3 was not affected by the expression of other known cofactors except thyroid receptor-associated protein (TRAP) 220/MED1 [29], [33]. This system revealed that unliganded TR without Pit1 and GATA2 is not the activator of the TSHβ gene [29]. We found that a putative nTRE/AP-1-like sequence is dispensable for T3-dependent inhibition [33] and proposed a model in which T3-bound TR (T3/TR) interferes with the transactivation function of GATA2, resulting in negative regulation of the TSHβ gene [33]. In this regard, the role of unliganded TR in the TRH signaling pathway should be re-considered.

Whereas Charles et al. [35] reported that TSHβ expression in pituitary-specific GATA2 knockout mice was not increased in the hypothyroid condition, where hypothalamic TRH secretion is increased, the functional relevance of GATA2 in TRH signaling was not clarified. To exclude the influence of negative feedback regulation by T3 on TRH production, we employed reporter assays in cultured cells. We demonstrate here that GATA2, but not Pit1 or unliganded TR, is the principal mediator of the TRH signal in transcriptional activation of the TSHβ gene.

## Materials and Methods

### Plasmid constructions

Because T3/TR has a tendency to suppress the firefly luciferase-based reporter gene [29], [33], we employed the CAT- and modified Renilla luciferase (hRluc)-based reporter systems (Promega, Madison, WI, USA). The human TSHβ promoters encompassing nt. −1193/+37 and nt. −128 to +37 were fused with the CAT gene, generating TSHβ(−1193/+37)-CAT and TSHβ(−128/+37)-CAT, respectively [29]. In these reporter plasmids, the pUC-derived AP-1 site [29], which may mediate PKC signal, was deleted from the original plasmid [22], [26], [27]. TSHβ-M1-mXmYmZ-CAT, TSHβ-M3-CAT, TSHβ(−128/+37)-hRluc and TSHβ-mP-M1-hRluc were reported elsewhere [31], [33]. The downstream GATA-RE in TSHβ(−128/+37)-hRluc was mutated from AGATAA to AGATCA using site-directed mutagenesis (Stratagene, La Jolla, CA), generating TSHβ(−128/+37)-G2C-hRluc. Expression plasmids for human Pit1 (pCB^6^-hPit1), mouse GATA2 (pcDNA3-mGATA2), and the deletion constructs of GATA2 (GATA2-NZ, GATA2-ZC, and GATA2-Zf) have been described elsewhere [33]. All mutated sequences and subcloning sites were confirmed by sequencing.

### Cell culture and transient transfection

CV1 [32] and GH3 [12] cells were grown in monolayer culture at 37 C under CO_2_/air (1:19) in Dulbecco's modified Eagle's medium (DMEM) containing 10% (v/v) fetal calf serum (FCS), penicillin G (100 units/ml), and streptomycin (100 µg/ml). TαT1 cells, a thyrotroph cell line from the mouse pituitary [36], were seeded on Matrigel-coated plates (Becton Dickinson Labware, Bedford, MA, U.S.A.) and maintained under the same conditions as CV1 and GH3 cells. All cells were trypsinized and plated in 6-well plates for 24 h before transient transfection. CV1 cells at a density of 2×10^5^ cells per well were transfected using the calcium phosphate technique, as described previously [37]. GH3 and TαT1 cells at a density of 6×10^5^ and 3×10^5^ cells per well were transfected using lipofectamine reagent (Promega) according to the manufacturer's protocol. After cells had been exposed to calcium phosphate/DNA precipitates or lipofectamine reagent for 20 or 5 h, the medium was replaced with fresh DMEM containing 5% fetal calf serum depleted of thyroid hormone [37] or medium supplemented with TRH, tetradecanoylphorbol acetate (TPA) or forskolin. After incubation for an additional 24 h, cells were harvested. CAT and β-galactosidase activities were measured as described previously [37]. Luciferase activities were measured with the Luciferase Assay System or Renilla Luciferase Assay System (Promega) using a Lumicounter 700 (Microtech Nichi-on, Chiba, Japan) [31], [33]. Protein concentration was determined by OD280. For each reporter assay, we performed transfection with pCMV-CAT, pGL4.13[luc2/SV40], or pGL4.74[hRLuc/TK], the magnitudes of which were adjusted to a value of 100%.

### Western blot

To assess the expression level of wild-type GATA2 and C349A mutant, CV1 cells in a 10 cm dish were transfected with an equal amount (10 µg/dish) of expression plasmids for wild-type or mutant GATA2s. After incubation for an additional 24 h, cells were harvested and whole cell extracts were fractionated by SDS-PAGE and subjected to Western blot with anti-GATA2 antibody (a kind gift from Dr. Takashi Minami, University of Tokyo).

### Gel shift assay

PG-probe (wild-type TSHβ sense; 5′-cagtatgaattttcaatagatgcttttcagataagaaa-3′ and antisense; 5′-tttcttatctgaaaagcatctattgaaaattcatactg-3′) were labeled with γ-^32^P-ATP using T4 polynucleotide kinase (Toyobo, Tokyo, Japan). CV1 cells were transfected with pcDNA3-mGATA2 (5 µg per 10 cm dish). After incubation with TRH or TPA, cells were harvested. Nuclear extracts (NEs) from CV1 cells were prepared as described previously [31]. The γ-^32^P-labeled probes and 2 µg NEs from transfected CV1 cells were incubated for 30 min on ice in 20 µl binding buffer containing 10 mM Tris-HCl (pH 7.6), 50 mM KCl, 0.05 mM EDTA, 2.5 mM MgCl_2_, 8.5% glycerol, 1 mM dithiothreitol, 0.5 µg/ml poly (dI-dC), 0.1% TritonX-100, and 1 mg/ml nonfat dry milk. DNA–protein complexes were resolved by electrophoresis on a 5% polyacrylamide gel at 100 V for 80 min at room temperature. For the supershift assay, antibodies against GATA2 (SC-267; Santa Cruz, CA, USA) were added. The gel was dried and labeled bands were visualized using the BAS-1000 autoradiography system (Fuji Film, Tokyo, Japan).

### Statistical analysis

Each reporter assay was performed in duplicate more than three times, and each result is expressed as the mean ± S.D. Statistical significance was examined by ANOVA and Fisher's protected least significant difference test using StatView 4.0 software (Abacus Concepts, Berkeley, CA, USA). P<0.05 was considered significant.

## Results

### TRH signaling enhances the transactivation of Pit1 and GATA2-dependent activity of the TSHβ gene in CV1 cells

**Figure 1:**
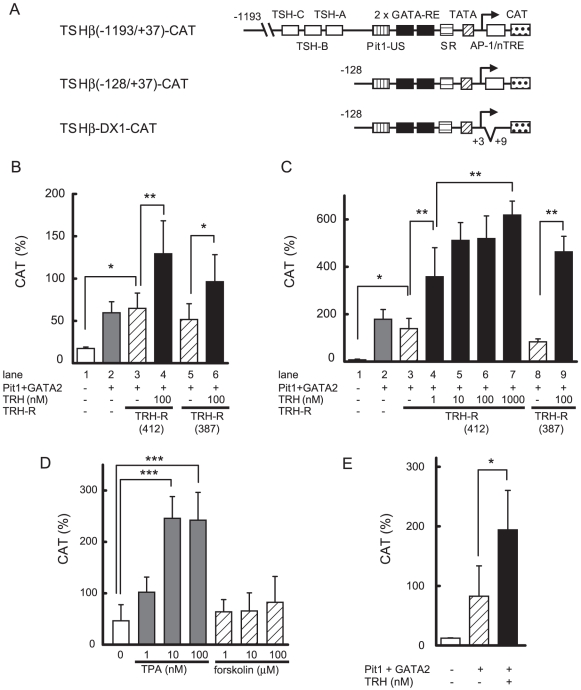
TRH/TRH-R1 stimulates GATA2/Pit1-dependent activity of the human TSHβ gene. (A) Schematics of TSHβ(−1193/+37)-, TSHβ(−128/+37)- and TSHβ-DX1-CAT. Putative TRH-responsive elements (TSH-A, nt. −274/−258 and TSH-C, nt. −402/−384) and PKA-sensitive element (TSH-B, nt. −333/−325) reported in the rat TSHβ gene are indicated as boxes in TSHβ(−1193/+37)-CAT. Pit1-US, a functional Pit1 binding site upstream of GATA-REs. GATA-REs, GATA responsive elements. SR, suppressor region. (B) and (C) Two µg TSHβ(−1193/+37)-CAT (B) or TSHβ(−128/+37)-CAT (C) was cotransfected into CV1 cells together with the expression plasmids for Pit1 (0.1 µg), GATA2 (0.2 µg), TRH-R1(412) or TRH-R1(387) (0.2 µg) and β-galactosidase-based reporter gene (pCH111, 1.1 µg). After 24h of culture, the cells were treated with 1–1000 nM TRH. (D) TPA, but not forskolin, enhances the Pit1/GATA2-dependent activity of TSHβ(−128/+37)-CAT. CV1 cells were cotransfected with 2.0 µg of TSHβ(−128/+37)-CAT together with the expression plasmids for Pit1 (0.1 µg), GATA2 (0.2 µg), TRH-R1 (0.2 µg; 412 aa or 387 aa) and pCH111 (1.1 µg). After 24h of culture, the cells were treated with 1–100 nM TPA or 1–100 µM forskolin. (E) AP-1-like sequence overlapping with putative nTRE is dispensable for TRH-induced activation of the TSHβ gene. Reporter assay with TSHβ-DX1-CAT was performed as described in (C). CAT activity was normalized with β-galactosidase activity. CAT activity for pCMV-CAT (5 ng/well) was taken as 100%. Data are expressed as the mean ± S.D. of at least three independent experiments. *, P<0.05; **, P<0.01; ***, P<0.001.

The human TSHβ promoter encompassing nt. −1193/+37 was fused to the CAT gene to generate TSHβ(−1193/+37)-CAT (Fig. 1A). As reported previously [29], co-expression of GATA2 and Pit1 activated TSHβ(−1193/+37)-CAT in CV1 cells (Fig. 1B, lane 1 and 2). The TRH-R1 gene generates long (412 aa) and short (387 aa) isoforms through alternative splicing of pre-mRNA [8]. In the presence of the long isoform, treatment with 100 nM TRH showed a further increase of promoter activity (Fig. 1B, lane 3 and 4). As shown in lane 5 and 6, the liganded short isoform elicited comparable potentiation. Since both TRH-R1s had similar effects and the long isoform is dominantly expressed in pituitary tissue [38], we employed it for further studies. It was reported that, in TtT97 TSHoma cells, the DNA sequence between nt. −271/−80 in the mouse TSHβ promoter (corresponding to sequence nt −269/−78 in the human gene) is sufficient for maximal promoter activity [39]. As shown in Fig. 1A, this region harbors a functional Pit1 binding site (Pit1-US) and two GATA-responsive elements (GATA-REs) [30]. These sequences are perfectly conserved in rodents and humans. We tested the effect of TRH/TRH-R1 on TSHβ(−128/+37)-CAT, which contains all of these elements (Fig. 1A), and again found that GATA2/Pit1-dependent activity (Fig. 1C, lane 1 and 2) was potentiated by TRH-R1 in the presence of 100 nM TRH (lane 3 and 6), suggesting that the TRH signal can be mediated via the region, nt −128/+37, containing Pit1-US and GATA-REs. The effect of TRH was dose-dependent (lane 3–7).

### Effect of TRH/TRH-R1 on Pit/GATA2-dependent activation of the TSHβ promoter is mediated by PKC but not PKA

Activation of the PKC signal by TRH/TRH-R1 is known to play a critical role in the expression of the PRL gene [9] as well as the TSHβ gene [13]. When CV1 cells were cotransfected with GATA2 and Pit1, the activity of TSHβ(−128/+37)-CAT was enhanced by the PKC activator, tetradecanoylphorbol acetate (TPA) (Fig. 1D). Although a cAMP-sensitive region (TSH-B) in the rat TSHβ promoter was reported at nt. −333/−325 [13], TRH-induced activity in the presence of Pit1 and GATA2 was maintained in TSHβ(−128/+37)-CAT (Fig. 1C, lane 3 and 4), which lacks the sequence corresponding to TSH-B (nt −363/−355 in the human genome, Fig. 1A). Thus, the stimulatory effect of TRH via Pit1 and GATA2 is independent of TSH-B. Whereas the PKA pathway may activate the TSHβ promoter via the interaction of Pit1 with CBP [40], the effect of forskolin on the Pit1/GATA2-dependent activity of the TSHβ gene was much smaller than that of TPA (Fig. 1D). The putative nTRE [27] in human TSHβ promoter overlaps an AP-1-like sequence (nt. −1/+6) [26]. To explore the involvement of this sequence in activation by TRH/TRH-R1, we tested a mutant reporter gene, TSHβ-DX1-CAT [33], where nTRE and AP-1-like sequences were deleted (Fig. 1A). Transactivation by TRH was not affected in this construct (Fig. 1E), suggesting that the reported nTRE or AP-1-like sequence is not required for the activity of the TSHβ promoter when it is stimulated by TRH via Pit1 and GATA2.

### TRH signaling potentiates the transactivating function of GATA2

**Figure 2:**
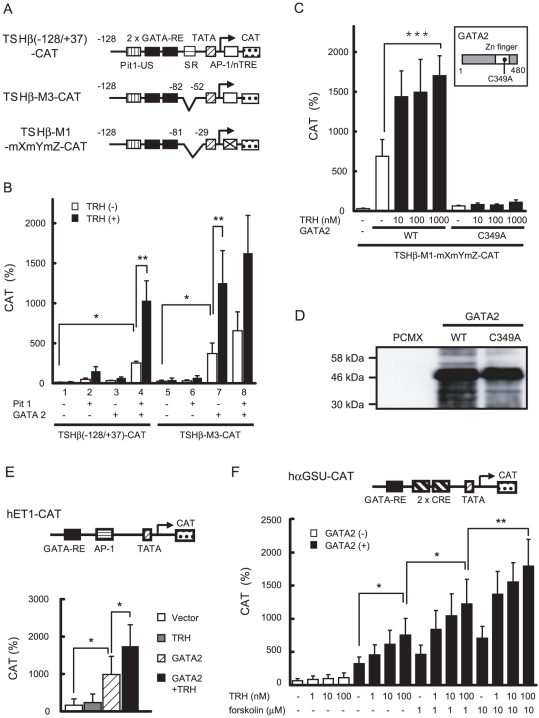
GATA2 alone mediates TRH signaling for transcription of the SR-less TSHβ promoter. (A) Schematics of TSHβ(−128/+37)-CAT, TSHβ-M3-CAT and TSHβ-M1-mXmYmZ-CAT. SR was deleted in both constructs and AP-1-like sequence/nTRE was mutated in TSHβ-M1-mXmYmZ-CAT. (B) and (C) Two µg TSHβ(−128/+37)-CAT, TSHβ-M3-CAT (B) and TSHβ-M1-mXmYmZ-CAT (C) were transfected into CV1 cells along with the expression plasmids for Pit1 (0.1 µg), GATA2 (0.2 µg), TRH-R1(412) (0.2 µg), and pCH111 (1.1 µg) in the presence or absence of 100 nM TRH. Basal and TRH-induced activities of TSHβ-M1-mXmYmZ-CAT were abolished with mutant GATA2, C349A (inset). (D) Expression level of wild-type GATA2 and C349A mutant. CV1 cells in 10 cm dish were transfected with an equal amount (10 µg/dish) of expression plasmids for wild-type or mutant GATA2s. Whole cell extracts were fractionated by SDS-PAGE and subjected to Western blot with anti-GATA2 antibody. (E) Schematic representation of hET1-CAT (upper panel). Two µg of hET1-CAT was transfected into CV1 cells along with expression plasmids for GATA2 (0.2 µg), TRH-R1(412) (0.2 µg), and pCH111 (1.1 µg) in the presence or absence of 100 nM TRH (lower panel). (F) Schematic representation of hαGSU-CAT (upper panel). Reporter assay was carried out as described in (E) in the presence or absence of 0–10 µM forskolin (lower panel). CAT activity was normalized with β-galactosidase activity. CAT activity for pCMV-CAT (5 ng/well) was taken as 100%. Data are expressed as the mean ± S.D. of at least three independent experiments. *, P<0.05; **, P<0.01.

We reported that the true transcriptional activator of the TSHβ gene is GATA2, while Pit1 protects GATA2 function from inhibition by the suppressor region (SR), which locates the 3′-flanking region of GATA-RE (Figs. 1A, 2A) [31]. In accordance with this, GATA2 alone can transactivate the SR-deleted construct, TSHβ-M3-CAT (Fig. 2B, lane 7). Notably, this activity was enhanced by TRH/TRH-R1. An identical result was found with TSHβ-M1-mXmYmZ-CAT (Fig. 2C), which contains only Pit1-US and GATA-REs (Fig. 2A). This construct was not activated by the mutant GATA2, C349A (Fig. 2C, inset), the DNA binding activity of which is disrupted [33]. Moreover, C349A was resistant to stimulation by TRH/TRH-R1 (Fig. 2C). The expression level of C349A was comparable with that of wild-type GATA2 (Fig. 2D). Thus, transactivation by GATA2 is important for not only basal but also TRH-induced activities of the TSHβ gene. As shown in Fig. 2E (upper panel), GATA-RE was reported in the endothelin-1 (ET-1) promoter [41]. This promoter was activated by GATA2 without Pit1 and this activity was enhanced by TRH/TRH-R1 (Fig. 2E, lower panel). Pit1-independent activation was also observed in the human αGSU promoter, which has a typical GATA-RE [42] and is known to be stimulated by TRH in pituitary primary culture [4] and TPA [43], [44]. Because this promoter contains two CREs close to GATA-RE, there may be cross talk between TRH/TRH-R signaling and PKA pathway. The GATA2-dependent activity of the human αGSU promoter was enhanced by the treatment of forskolin in a dose dependent manner (Fig. 2F). The effect of co-stimulation with forskolin and TRH/TRH-R had additive but not synergistic effect.

### While inhibition by T3/TR is dominant over the activation by TRH, unliganded TR is not involved in TRH signaling

**Figure 3:**
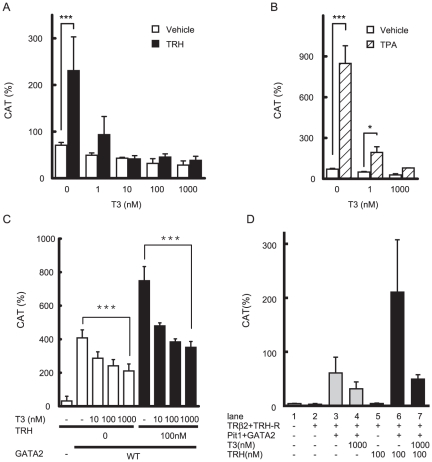
Negative regulation by T3/TRβ2 is dominant over the transcriptional activation induced by TRH/TRH-R1 or TPA. (A) and (B) Two µg TSHβ(−128/+37)-CAT was transfected into CV1 cells along with expression plasmids for Pit1 (0.1 µg), GATA2 (0.2 µg), TRH-R1(412) (0.2 µg), TRβ2 (0.2 µg) and pCH111 (1.1 µg). After 24 h of culture, the cells were treated with 100 nM TRH (A) or 10 nM TPA (B) for an additional 24 h in the presence or absence of 1–1000 nM T3. (C) Two µg TSHβ-M1-mXmYmZ-CAT (Fig. 2A) was transfected into CV1 cells along with expression plasmids for GATA2 (0.2 µg), TRH-R1(412) (0.2 µg), TRβ2 (0.2 µg), and pCH111 (1.1 µg). After 24 h of culture, the cells were treated with 100 nM TRH for an additional 24 h in the presence or absence of 1–1000 nM T3. (D) Unliganded TRβ2 alone does not mediate TRH signaling. TSHβ(−128/+37)-CAT and expression plasmids for human TRβ2 (0.2 µg), TRH-R1(412) (0.2 µg), and pCH111 β(1.1 µg) were transfected into CV1 cells along with or without the expression plasmids for Pit1 (0.1 µg) and GATA2 (0.2 µg). After 24 h of culture, the cells were treated with 100 nM TRH and/or 1000 nM T3 for an additional 24 h. CAT activity was normalized with β-galactosidase activity. CAT activity for pCMV-CAT (5 ng/well) was taken as 100%. Data are expressed as the mean ± S.D. of three independent experiments. *, P<0.05; **, P<0.01, ***, P<0.001.

Using CV1 cells transfected with the expression plasmids for TRH-R1, GATA2, Pit1 and pituitary-specific TR, TRβ2, we compared the potency of TRH-induced activation with T3/TR-dependent inhibition in the context of the TSHβ promoter. As shown above (Fig. 1C), 100 nM TRH is sufficient to enhance transactivation by Pit1 and GATA2, and this concentration is compatible with the previous reports using pituitary cells [4]. As we reported previously [29], [33], [34], T3/TRβ2 efficiently inhibited GATA2/Pit1-dependent transactivation of TSHβ(−128/+37)-CAT stimulated by 100 nM TRH (Fig. 3A). T3/TRβ2 also inhibited TPA-induced activity in the presence of Pit1 and GATA2 (Fig. 3B). TRH-induced activity of TSHβ-M1-mXmYmZ-CAT (Fig. 2A) with GATA2 was repressed by T3/TRβ2 (Fig. 3C), indicating that inhibition by T3/TRβ2 is dominant over TRH/GATA2-induced activation of the TSHβ promoter. If unliganded TR is a transcriptional activator of the TSHβ gene [22], it might play a role in transcriptional activation of the TSHβ gene by TRH [24], [27]. As shown in Fig. 3D, unliganded TRβ2 alone failed to stimulate basal or TRH-induced activity of the TSHβ promoter (lane 1, 2 and 5). Thus, unliganded TRβ2 without Pit1 or GATA2 is not a mediator of TRH signaling in TSHβ gene regulation.

### Zn finger domain of GATA2 plays a pivotal role in mediating TRH signaling

**Figure 4:**
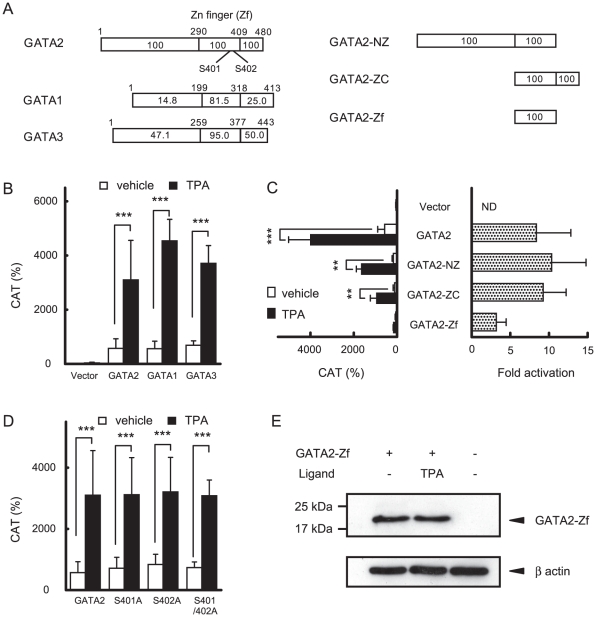
GATA2-Zf mediates TRH/PKC signaling for the TSHβ promoter. (A) Schematics of full-length GATA2, GATA1, GATA3, GATA2-NZ, ZC and Zf. Homology of amino acids between GATA3 and 1 relative to GATA2 is indicated as a percentage in boxes. (B) and (C) TSHβ-M1-mXmYmZ-CAT (2.0 µg, Fig. 2A), and pCH111 (1.1 µg) were cotransfected into CV1 cells with various GATA2 mutants (0.2 µg) with or without 10 nM TPA (left panel). CAT activity was normalized with β-galactosidase activity. (D) Alanine substitutions with serines at codons 401 or 402 in mouse GATA2-Zf did not affect TRH-induced activation. Reporter assay was carried out as described in (B) and (C). CAT activity for pCMV-CAT (5 ng/well) was taken as 100%. The fold activation (right panel) was calculated from CAT activity with TPA divided by that without TPA. ND, not determined. Data are expressed as the mean ± S.D. of at least three independent experiments. **, P<0.01; ***, P<0.001. (E) CV1 cells were cotransfected with FLAG-tagged GATA2 expression plasmid and were treated with 10 nM TPA. Whole cell extracts were resolved by SDS/PAGE and western blot analyses for GATA2-Zf and β-actin were performed using the anti-FLAG and anti-β-actin antibodies, respectively.

The Zn finger domain of GATA2 (GATA2-Zf) has high sequence homology with those of GATA1 and 3 (Fig. 4A, left panel) and GATA3 partially compensates for GATA2 in pituitary-specific GATA2-knockout mice [35]. As shown in Fig. 4B, TPA enhanced the transactivation of TSHβ-M1-mXmYmZ-CAT (Fig. 2A) by GATA2 as well as GATA1 and 3 without Pit1. While TRH signaling was abolished by the mutation (C349A) in GATA2-Zf (Fig. 2C), the transcriptional activity of the deletion constructs that lack the N- or C-terminal region of GATA2 (Fig 4A, right panel) was significantly stimulated by TPA (Fig. 4C), showing that GATA2-Zf is important for the activation by TRH/PKC signaling. Although phosphorylation of serine residue at codon 401 in human GATA2 was reported to increase its nuclear localization [45], substitution of the serines at codon 401 and 402 in the mouse GATA2 (Fig. 4A, left panel) with alanines did not affect the response to TPA (Fig. 4D). As shown in Fig. 4E, TPA (10 nM) treatment did not alter the molecular weight of FLAG-tagged GATA2-Zf expressed in CV1 cells.

### TRH signaling facilitates the DNA binding capacity of GATA2

**Figure 5:**
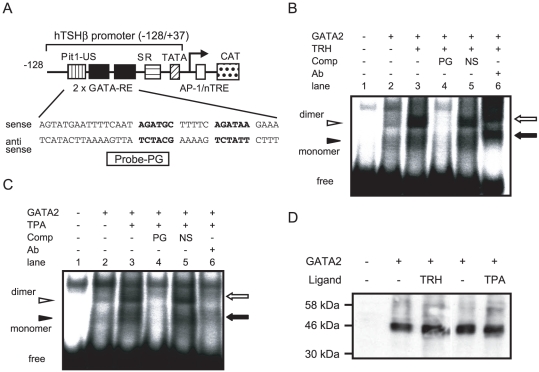
Treatment with TRH and TPA enhance DNA binding of GATA2. (A) Schematic of probe-PG that has two GATA-REs. (B) and (C) CV1 cells were cotransfected with GATA2 and TRH-R1(412) expression plasmids and were treated with 100 nM TRH (B) or 10 nM TPA (C). NE from CV1 cells (2 µg) was incubated with γ-^32^P-labeled probe-PG. DNA-protein complexes were resolved by electrophoresis on a 5% polyacrylamide gel. Solid arrowhead, GATA2 monomer; open arrowhead, GATA2 dimer. Solid and open arrows on the right indicate the supershifts of GATA2 monomer and dimer, respectively. Ab, anti-GATA2 antibody; PG, cold probe-PG; NS, non-specific oligonucleotide; free, free radiolabeled probe-PG. (D) Western blot analysis was performed using the anti-GATA2 antibody. NEs of CV1 cells (10 µg) described in (B) and (C) were resolved by SDS/PAGE (10% gel). Molecular weight markers are shown on the left.

Because GATA2-Zf functions as an interface to recognize GATA-REs, we performed a gel shift assay to assess the effect of TRH on DNA binding of GATA2-Zf. CV1 cells were co-transfected with TRH-R1 and GATA2, and cultured with or without 100 nM TRH. The nuclear extracts were incubated with ^32^P-labeled DNA fragment harboring two GATA-REs in the TSHβ promoter (Fig. 5A). As we reported [31], two bands corresponding to GATA2 monomer and dimer were observed (Fig. 5B, lane 2). Interestingly, these bands were potentiated by TRH treatment (lane 3). These signals were abolished by the 50-fold specific competitor (lane 4) but not unrelated DNA (lane 5) and supershifted by a specific antibody against GATA2 (lane 6). Identical results were obtained in the nuclear extract from CV1 cells cultured with TPA (Fig. 5C). The expression level of GATA2 in CV1 cells was not affected by treatment with TRH or TPA (Fig. 5D). Collectively, TRH/PKC signal facilitates the DNA binding of GATA2 in the TSHβ promoter.

### TRH signaling in somato-lactotroph cell line, GH3, transfected with GATA2 gene

**Figure 6:**
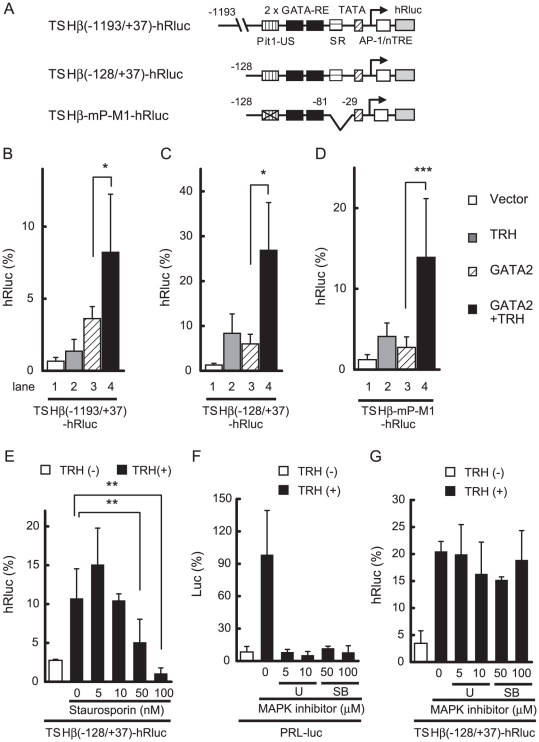
Pit1-US is dispensable for TRH signaling in the SR-less TSHβ promoter in pituitary-derived GH3 cells transfected with GATA2. (A) Schematics of TSHβ(−1193/+37)-hRluc, TSHβ(−128/+37)-hRluc, and TSHβ-mP-M1-hRluc. In TSHβ-mP-M1-hRluc, Pit1-US was mutated and SR was deleted. (B) and (C) Co-transfection of GATA2 allows TRH/TRH-R1 to activate TSHβ(−1193/+37)-hRluc (B) and TSHβ(−128/+37)-hRluc (C). (D) Although Pit1-US is mutated in TSHβ-mP-M1-hRluc, deletion of SR enabled GATA2 to activate transcription. Using lipofection, 0.4 µg TSHβ(−1193/+37)-hRluc (B), TSHβ(−128/+37)-hRluc (C), or TSHβ-mP-M1-hRluc (D) was cotransfected into GH3 cells together with the expression plasmid for GATA2 (0.05 µg) and pGL4.13[luc2/SV40] (0.05 µg). After 5 h of culture, the cells were treated with 100 nM TRH for 12 h. hRluc activity was normalized with firefly luciferase activity of pGL4.13[luc2/SV40]. (E) GH3 cells were cotransfected with TSHβ(−128/+37)-hRluc (0.45 µg) and GATA2 expression plasmid (0.05 µg). After 5 h culture, cells were treated with 100 nM TRH for 12 h in the presence or absence of PKC inhibitor, staurosporin (5–100 nM). hRluc activity was normalized with protein concentration. (F) GH3 cells were cotransfected with PRL-luc plasmid (0.5 µg) and treated with 100 nM TRH for 12 h with or without MAPK inhibitor, U0126 (5–10 µM) or SB202190 (50–100 µM). Firefly luciferase activity was normalized with protein concentration. (G) TRH/GATA2-induced activation of the TSHβ promoter depends on PKC but not MAPK in GH3 cells. GH3 cells were cotransfected as described in (E). After 5 h culture, the cells were treated with 100 nM TRH for 12 h with or without U0126 (5–10 µM), or SB202190 (50–100 µM). The magnitude of hRluc activity for pGL4.74 [hRLuc/TK] or firefly luciferase activity for pGL4.13 [luc2/SV40] (50 ng/well) was taken as 100%. Data are expressed as the mean ± S.D. of at least three independent experiments. U, U0126; SB, SB202190; *, P<0.05; **, P<0.01; ***, P<0.001.

GH3 is a somato-lactotroph cell line expressing endogenous TRH-R1(412) [8] and Pit1 but not GATA2 [30]. In this regard, GH3 cells provide a suitable experimental system to explore the function of GATA2. Because of the low transfection efficiency of GH3 cells, we fused the hRluc-based reporter gene with human TSHβ promoters to generate TSHβ(−1193/+37)- or TSHβ(−128/+37)-hRluc (Fig. 6A). Although GH3 cells express endogenous Pit1, the transcriptional activity of TSHβ(−1193/+37)-hRluc was very low (Fig. 6B, lane 1), presumably due to the lack of GATA2. While TRH treatment modestly activated the TSHβ promoter in GH3 cells via endogenous TRH-R1 (lane 2) [40], transcription in the presence of GATA2 was further enhanced by TRH treatment (lane 3 and 4). In TSHβ(−128/+37)-hRluc, GATA2-dependent activation was also potentiated by TRH (Fig. 6C, lane 3 and 4). Although disruption of Pit1-US is known to abolish the activity of the TSHβ promoter [31], [46], deletion of SR enables GATA2 to re-activate transcription without Pit1 [31]. As predicted, TRH-induced GATA2 activity was maintained in TSHβ-mP-M1-hRluc, where Pit1-US was mutated and SR was deleted (Fig. 6A and D). In TSHβ(−128/+37)-hRluc, TRH induced some activity without co-expression of GATA2 (Fig. 6C, lane 1 and 2), but Pit1 is unlikely to mediate this activity since similar TRH-induced activity was observed in TSHβ-mP-M1-hRluc (Fig. 6D), where a functional Pit binding site, Pit1-US, was mutated. These results suggest again that Pit1 is not the direct target of TRH signaling.

### TRH/GATA2-induced transactivation of TSHβ gene is independent of MAPK signaling

As shown in Fig. 6E, a PKC inhibitor, staurosporin, reduced the TRH-induced activity of TSHβ(−128/+37)-hRluc in GH3 cells co-transfected with GATA2. In this cell line, PKC is known to stimulate the mitogen-activated protein kinase (MAPK) pathway [9], resulting in activation of the PRL promoter via the Ets family transcription factor [47]. Consistently, TRH treatment stimulated the activity of PRL-Luc, where the PRL promoter was fused with the luciferase gene (Fig. 6F). We speculated that the role of MAPK may be different between the TSHβ gene and the PRL gene if TRH signaling in the former gene is mediated by GATA2 but not Ets. While MAPK inhibitors, U0126 and SB202190, abolished the stimulatory effect of TRH on PRL-Luc (Fig. 6F), the TRH-induced activity of GATA2 in TSHβ(−128/+37)-hRluc was resistant to these compounds (Fig. 6G). Similar results were observed when we used a c-Jun N-terminal kinase inhibitor, SP600125 (data not shown).

### GATA2 is the limiting factor for the TRH signaling in thyrotroph cell line, TαT1

**Figure 7:**
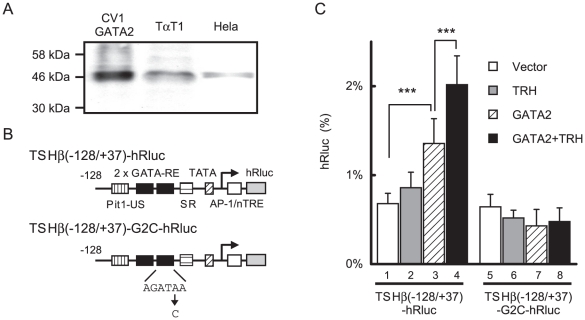
GATA2 is a limiting factor for TRH-induced transactivation of the TSHβ gene in TαT1 cells. (A) Expression of endogenous GATA2 in thyrotroph-derived cell line, TαT1 is comparable with that of Hela cells. Whole cell extracts of CV1 cells transfected with GATA2 expression plasmid (lane 1), TαT1 cells (lane 2) and Hela cells (lane 3) were fractionated by SDS-PAGE and subjected to Western blot with anti-GATA2 antibody. (B) Schematic representations of TSHβ(−128/+37)-hRluc and TSHβ(−128/+37)-G2C-hRluc. Downstream GATA-RE was mutated in TSHβ(−128/+37)-G2C-hRluc. (C) Co-transfection of GATA2 enables TRH to activate the TSHβ promoter (lane 1–4). GATA2-dependent as well as TRH-induced transcriptional activations were abolished by the mutation of GATA-RE (lane 5–8). TαT1 cells were cotransfected with TSHβ(−128/+37)-hRluc (0.45 µg) or TSHβ(−128/+37)-G2C-hRluc along with GATA2 expression plasmid (0.05 µg). After 5 h culture, the cells were treated with 100 nM TRH for 12 h. hRluc activity was normalized with the activity of pCMV-βgal. The magnitude of hRluc activity for pGL4.74 [hRLuc/TK] (50 ng/well) was taken as 100%. Data are expressed as the mean ± S.D. of at least three independent experiments. ***, P<0.005.

TαT1 cells were regarded as a thyrotroph cell line [48] and reported to express endogenous Pit1, TRβ2 [36] and (presumably) TRH-R [49]. Western blotting with anti-GATA2 antibody revealed that the expression level of GATA2 in this cell line is low and similar to that in Hela cells (Fig. 7A)[50]. We previously found that, in Hela cells, co-expression of GATA2 as well as Pit1 was required for the reporter assay with TSHβ(−128/+37)-CAT (data not shown). In a reporter assay with TSHβ(−128/+37)-hRluc (Fig. 7B) in TαT1 cells, the effect of 100 nM TRH treatment exhibited minimal induction (Fig. 7C, lane 2) and transient transfection of GATA2 permitted significant enhancement by TRH (Fig. 7C, lane 3 and 4). This suggested that GATA2 is a limiting factor for TRH signaling. We tested TSHβ-G2C-hRluc, where downstream GATA-RE was mutated (Fig. 7B). Although this construct harbors intact Pit1-US, induction by TRH was abolished (Fig. 7C, lane 7 and 8), suggesting that GATA2 is essential for TRH-induced activation of the TSHβ gene in TαT1 cells.

## Discussion

We reported previously that the true transcriptional activator of the TSHβ gene is GATA2, not Pit1 [31] or unliganded TR [29]. Here, we demonstrated that GATA2 is the principal mediator of TRH signaling for TSHβ expression. TRH-induced activity is abolished by amino acid substitution in the GATA2-Zn finger domain (C349A, Fig. 2C). TRH signal stimulates GATA2-dependent activities of the αGSU gene and the ET-1 gene (Fig. 2E and F). In agreement with previous reports [17]–[19], the current work provides additional lines of evidence that the Pit1 or Pit1-CBP/p300 complex is not the main target of TRH signaling. First, GATA2 is sufficient and Pit1 is dispensable for TRH-induced activation of the TSHβ promoter when SR was deleted (Fig. 2B and 6D). Second, Pit1 in TαT1 cells [36] failed to mediate the TRH signal in TSHβ-G2C-hRluc, which has intact Pit1-US but harbors a mutation in GATA-RE (Fig. 7B and C). Third, although Pit1 may be phosphorylated [15], [16], [20], substitution of candidate amino acids with alanines did not affect transcriptional activity of the TSHβ gene (data not shown), as in the PRL gene [21], [47]. We reported that the function of Pit1 does not have conventional synergism with GATA2, but rather protects GATA2 function from SR-induced suppression (de-repression).

Kim et al. [49] reported that the TRH signal may induce the expression of a transcription factor, Lhx2, in TαT1 cells. According to the authors, Lhx2 subsequently binds the DNA sequence (nt. −86/−68 in the rat TSHβ gene) that overlaps with SR, and activates the TSHβ promoter even in αTSH cells, which lack endogenous Pit1 [17], [49]; however, their findings were not compatible with reduced TSHβ expression in a CPHD patient with mutant Pit1 [11]. Although they reported that Lhx2 also activates the TSHβ promoter in GH3 cells, which lacks endogenous GATA2, this does not agree with our finding that the mutation of GATA-RE abolished the transactivation by TRH in TαT1 cells (Fig. 7C). We could not detect the activation of the human TSHβ promoter (TSHβ(−128/+37)-CAT) by co-transfection of the Lhx2 expression plasmid into CV1 cells (data not shown).

TRH/TRH-R-induced PKC activity is known to stimulate MAPK activity [9], [51] and MAPK subsequently phosphorylates Ets transcription factors and activates the PRL gene [47]. Of note, phosphorylation by MAPK does not affect the functions of GATA2 [52] and the transactivation domain of Pit1 [53]. The current study using GH3 cells transfected with GATA2 indicates that TRH/GATA2/Pit1-induced activation of the TSHβ promoter depends on PKC (Fig. 6E) but not MAPK (Fig. 6G). To our knowledge, this is the first report showing the difference in the TRH signaling pathway between the TSHβ gene and the PRL gene. Whereas cAMP is known to activate the TSHβ gene [5], [13] and Pit1-CBP/p300 complex plays a role in TSHβ expression [23], [31], [40], activation of GATA2 by TRH is independent of the PKA pathway because transactivation of the TSHβ promoter in the presence of Pit1 and GATA2 was not enhanced by forskolin (Fig. 1D) or did not require a putative cAMP-sensitive site, TSH-B (Fig. 1C). This is in agreement with previous in vivo studies [8], [51], [54], [55].

**Figure 8:**
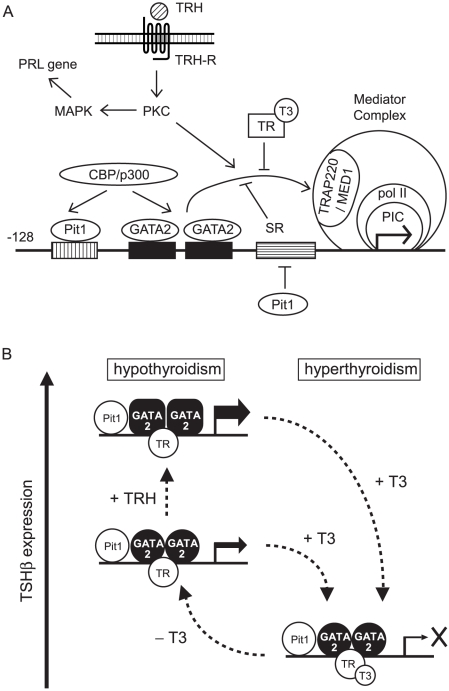
Mechanism of TRH-induced transactivation of the TSHβ gene. (A) GATA2 plays a pivotal role in both TRH-induced activation as well as T3/TR-dependent inhibition. Unlike the PRL gene, transactivation of the TSHβ gene is independent of the MAPK pathway. Pit1 competes the binding of SR binding protein and protects the function of GATA2 from inhibition and also binds to Pit1-US to cooperate with GATA2s to recruit a coactivator, CBP/p300. PIC, pre-initiation complex. Pol II, RNA polymerase II. (B) Roles of GATA2, Pit1 and TR in hypo- and hyper-thyroidism. Co-expression of GATA2 and Pit1 determines thyrotroph differentiation. With support by Pit1, GATA2 maintains the basal transcription of the TSHβ gene and mediates TRH/TRH-R1 signaling in hypothyroidism while unliganded TR alone is not a transcriptional activator. Inhibition by T3/TR is dominant over the activation by GATA2 even in the presence of TRH signaling.

GATA2-NZ and ZC partially mediate TRH-induced transactivation (Fig. 4C) while mutation of GATA2 (C349A) abolished it (Fig. 2C), suggesting the crucial role of GATA2-Zf in TRH signaling. Although the detailed mechanism of how GATA2-Zf mediates TRH signaling is unknown, there are the following possibilities. First, stimulation by TRH/TRH-R1 or TPA enhanced the DNA binding capacity of GATA2, as shown in our gel shift assay (Fig. 5). Similar effects of PKC were reported in the GATA-REs of vascular adhesion molecule-1 promoter [56] and in the human αGSU gene [43]. Second, phosphorylation of serine at codon 401 in GATA2-Zf (Fig. 4A) may facilitate the nuclear localization of GATA2 [45]. In our experimental condition, however, the substitution of serines at codon 401 or 402 with alanines did not cripple the TPA effect (Fig. 4D). As reported previously [57], the effect of the phosphorylation of these serine residues may be specific to the promoter context. Finally, TRAP220/MED1 plays an important role (Fig. 8A). TSHβ expression was reduced in heterozygous TRAP220/MED1-deficient mice [58] and GATA2-Zf interacted with TRAP220/MED1 [59]. Our preliminary data showed that GATA2-dependent activation of the TSHβ promoter by TRH signaling is also mediated by TRAP220/MED1 (data not shown). Because TRAP220/MED1 contains multiple amino acid sequences which can be phosphorylated by various kinases, including PKC [60], [61], its phosphorylation may be involved in the TRH signaling pathway.

Unexpectedly, we found that the GATA2 expression level in TαT1 cells is insufficient for TRH-induced activation (Fig. 7C) and failed to detect in vivo binding of GATA2 to the TSHβ promoter in this cell line (data not shown). Although TαT1 was established from transgenic mice harboring SV40-large T antigen fused with the αGSU promoter [36], [48], high and sustained expression of GATA2 may increase the amount of SV40-large T antigen (Fig. 2F), resulting in phenotype alteration with limited GATA2 expression. Forced expression of GATA2 in TαT1 cells enabled the wild-type TSHβ gene to be activated by TRH stimulation (Fig. 7C, left panel), indicating that GATA2 is a limiting factor for the TRH signaling in this cell line.

Although genetic ablation of H-P-T axis-related genes has been tested [24], [28], [62] and pituitary-specific GATA2-knockout mice established [35], the relative contributions of TRH signaling and negative feedback by T3 in TSHβ expression have not been clarified at the molecular level. We found that T3/TRβ2 rigorously inhibits GATA2-dependent activity enhanced by TRH or TPA (Fig. 3), suggesting that, with high T3 concentration, repression by T3/TR is dominant over the effect of TRH signaling (Fig. 8B). This is compatible with the recent report comparing Pax8-null mice with Pax8/TRH-R1 double knockout mice [62] and the previous study that administration of TRH failed to stimulate TSHβ synthesis in a subject with overt thyrotoxicosis [63]. Our prior report suggests that T3-dependent interaction of TR with TRAP220/MED1 may interfere with the function of GATA2 [33] (Fig. 8A). TRAP220/MED1 is a constituent of the Mediator complex that directly regulates the function of RNA polymerase II (pol II) [64]. Given that inhibition by T3 targets the final process of GATA2-induced transactivation, i.e. TRAP220/MED1-pol II complex, repression might occur downstream of or in the same step as TRH-induced activation.

It should be noted that release of the suppression by T3/TR is necessary but not sufficient for TSHβ expression. In hypothyroidism, the majority of TRβ2 in thyrotroph is unliganded and TRH production in the hypothalamus is increased [65] (Fig. 8B). Nikrodhanond et al. [24] compared TSH production among wild-type, TRβ-knockout and TRH/TRβ-double knockout mice under hypothyroid conditions and made two important findings. First, TRβ-knockout mice and wild-type mice exhibited comparable TSHβ expression in hypothyroidism where TRH secretion from the hypothalamus is elevated [65]. This supports our finding that unliganded TRβ is not essential for TRH-induced TSHβ expression (Fig. 3D, lane 2 and 5). Second, TSH level in hypothyroidism was severely impaired in TRH/TRβ-double knockout mice compared with wild-type or TRβ-knockout mice, suggesting that elevation of TSHβ expression requires the TRH signal even when inhibition by T3/TR is released (Fig. 8B). We found here that the factor mediating TRH signal for TSHβ expression is GATA2 (Fig. 2C, 3D and 7C). This is supported by the report that pituitary-specific GATA2-knockout mice exhibit reduced TSHβ expression in hypothyroidism, where stimulation by TRH should be elevated [35]. As shown in Fig. 8B, GATA2 mediates not only the negative regulation by T3/TR [33] but also TRH-induced activation of the TSHβ gene.
